# Antimalarial drug resistance in the Central and Adamawa regions of Cameroon: Prevalence of mutations in *P*. *falciparum crt*, *Pfmdr1*, *Pfdhfr* and *Pfdhps* genes

**DOI:** 10.1371/journal.pone.0256343

**Published:** 2021-08-19

**Authors:** Aline Gaelle Bouopda Tuedom, Elangwe Milo Sarah-Matio, Carole Else Eboumbou Moukoko, Brice Lionel Feufack-Donfack, Christelle Ngou Maffo, Albert Ngano Bayibeki, Hermann Parfait Awono-Ambene, Lawrence Ayong, Antoine Berry, Luc Abate, Isabelle Morlais, Sandrine Eveline Nsango

**Affiliations:** 1 Department of Biological Sciences, Faculty of Medicine and Pharmaceutical Sciences, University of Douala, Douala, Cameroon; 2 Malaria Research Unit, Centre Pasteur du Cameroun, Yaoundé, Cameroun; 3 UMR MIVEGEC, IRD, CNRS, Institut de Recherche pour le Développement, Université Montpellier, Montpellier Cedex, France; 4 CNRS UPR9022, INSERM U963, Strasbourg, France; 5 Université Catholique d’Afrique Centrale, Yaoundé-Campus Messa Cameroun, Yaoundé, Cameroun; 6 Laboratoire de Recherche sur le Paludisme, Organisation de Coordination pour la lutte contre les Endémies en Afrique Centrale (OCEAC), Yaoundé, Cameroun; 7 Service de Parasitologie-Mycologie, Centre Hospitalier Universitaire de Toulouse et UMR152 UPS-IRD, Université de Toulouse, Toulouse, France; Menzies School of Health Research, AUSTRALIA

## Abstract

The spread of *Plasmodium falciparum* resistant parasites remains one of the major challenges for malaria control and elimination in Sub Saharan Africa. Monitoring of molecular markers conferring resistance to different antimalarials is important to track the spread of resistant parasites and to optimize the therapeutic lifespan of current drugs. This study aimed to evaluate the prevalence of known mutations in the drug resistance genes *Pfcrt*, *Pfmdr1*, *Pfdhfr and Pfdhps* in two different epidemiological settings in Cameroon. Dried blood spots collected in 2018 and 2019 from asymptomatic individuals were used for DNA extraction and then the *Plasmodium* infection status was determined byPCR. Detection of SNPs was performed by nested PCR followed by allele-specific restriction analysis (ASRA). The prevalence of each genotype was compared between sites using the Chi square and Fisher’s exact tests. A high prevalence of the *Pfcrt* K76 wild type allele was found in both sites (88.5 and 62.29% respectively; P< 0,0001). The prevalence of *Pfmdr1* mutations 86Y and 1246Y was respectively 55.83 and 1.45% in Mfou and 45.87 and 5.97% in Tibati, with significant difference between the studied areas (P<0.0001). Overall, the *Pfdhfr* triple-mutant genotype (51I/59R/108N) was highly prevalent (> 96%), however no SNP was detected at codon 164. In *Pfdhps*, the prevalence of the 437G mutation reached (90%) and was at higher frequency in Mfou (P< 0.0001). Overall, the *Pfdhps* mutations 540E and 581G were less common (0.33 and 3.26%, respectively). The quadruple resistant genotype (*Pfdhfr* 51I/59R/108N+*Pfdhp*437G) was found almost 90% of the samples. The wild-type genotype (*Pfdhfr* N51/C59/S108/164I+*Pfdhps* A437/K540/A581) was never identified and the sextuple mutant (*Pfdhfr* 51I/59R/108N+*Pfdhp*437G/540E/581G), kwon as super resistant appeared in two samples from Tibati. These findings demonstrate declining trends in the prevalence of mutations conferring resistance to 4-aminoquinolines, especially to chloroquine. However, a high level of mutations in *P*. *falciparum* genes related to SP resistance was detected and this raises concerns about the future efficacy of IPTp-SP and SMC in Cameroon.

## Background

Despite global efforts to fight against malaria, the disease remains a serious public health problem. Latest estimations from WHO reported 229 million cases of malaria and 409.000 deaths worldwide in 2019, 94% of the cases were recorded in the WHO African Region where nearly 99% of malaria cases were caused by *Plasmodium falciparum* [1]. In Cameroon, the National Malaria Control Program (NMCP) indicated that, approximately 28% of morbidity and 18.3% of deaths were due to malaria in 2019 [2]. Unfortunately, disruption of malaria interventions due to COVID-19 will likely worsen the situation in the future [3]. Malaria case management has been compromised by parasite resistance to most antimalarial drugs, leading authorities to change guidelines for the treatment of malaria over time [4]. Indeed, selection pressure of drugs delivered as monotherapy drives the emergence and spread of antimalarial drug resistance. Chloroquine (CQ) resistance in *P*. *falciparum* was first detected in Colombia and Cambodia-Thailand border in the late 1950s after decades of heavy use and then resistance spread worldwide, reaching Africa in the late 1970s [5]. The same scenario then happened with sulfadoxine-pyrimethamine (SP), the antifolate drug that was used after withdrawal of CQ in the late 1990s. In 2001, WHO recommended the use of artemisinin-based combination therapies (ACTs) as first line treatment for uncomplicated *P*. *falciparum* malaria [6].

Cameroon’s authorities adopted the artesunate-amodiaquine (ASAQ) combination as first-line treatment of malaria in 2004 and artemether-lumefantrine (AL) as an alternative ACT in 2006 [7, 8]. In high malaria transmission areas, WHO recommends chemoprophylaxis as a strategy to prevent malaria, especially in risk groups [1]. Thus, SP continues to be used as an intermittent preventive treatment for prevention of malaria in pregnancy (IPTp-SP) [9, 10]. Also SP combined to amodiaquine (AQ) is used for seasonal malaria chemoprevention (SMC) in children within the age group 3–59 months in the North region of Cameroon where malaria transmission is seasonal [8]. The change in antimalarial policy has remarkably contributed to the reduction in mortality due to malaria in Cameroon [2, 11].

However, malaria case management and prevention remain highly threatened by the emergence and spread of drug resistance in *P*. *falciparum* parasites [12]. Presently, resistance has emerged against almost all antimalarial drugs, including the old molecules either administered as monotherapy, CQ and SP, or used as partner drugs in ACTs, amodiaquine (AQ), mefloquine (MQ) and lumefantrine (L) [13–15]. In the late 2000s, *P*. *falciparum* resistance to artemisinin (ART) was reported in South-East Asian regions particularly along the Cambodia–Thailand border, and this finding threatens worldwide initiatives and efforts to control malaria [16–19]. Fortunately, ART resistance has not been reported in Africa so far and treatments with ACTs remain effective [20–24]. However, polymorphisms in genes associated with resistance to partner drugs compromise the long-term efficacy of ACTs [21, 25, 26]. Moreover, resistance to ACT partner drugs emerged before that of artemisinin, thus monitoring the resistance level of partner drugs is a key element to maintain the effectiveness of ACTs [27].

Several studies have conclusively indicated a correlation between the development of antimalarial drug resistance and the presence of single nucleotide polymorphisms (SNPs) in *P*. *falciparum* parasite genes determining drug effects [28, 29]. For instance, the K76T mutation in the *P*. *falciparum* resistance transporter (*Pfcrt*) gene confers resistance to CQ [30]. Polymorphisms in the multi-drug resistance gene of *P*. *falciparum* (*Pfmdr1*), especially the N86Y, Y184F and D1246Y mutations [31], are linked with resistance against CQ, AQ, L, Quinine (Q) and Halofantrine (HL) [32]. It has been well documented that polymorphisms in the *Pfcrt* and *Pfmdr1* genes are currently being driven by *P*. *falciparum* susceptibility to ACT partner drugs [26, 27, 33]. Indeed, the combination of *Pfcrt* 76T and the *Pfmdr1* 86Y-184Y-1246Y triplet is linked to recrudescence and reinfection after treatment with artesunate-amodiaquine AS-AQ [27, 34]. An opposite pattern has been observed for artemether-lumefantrine (AL), with a strong selection of *Pfmdr1* N86 and *Pfcrt* K76 alleles in AL-treated patients who are reinfected [35]. SNPs in the dihydrofolate reductase (*dhfr*) [30, 36, 37] and dihydropteroate synthase (*dhps*) genes [38] are associated with SP resistance. Successive accumulation of mutations in both *Pdhfr* and *Pdhps* genes confers high levels of SP resistance and clinical treatment failure in several epidemiological settings [39, 40].

According to Naidoo *et al*, three combinations of SNPs in *Pdhfr* and *Pdhps* genes are used to assess the level of SP resistance in endemic areas [41]. The quadruple mutant (*Pfdhfr* N51I, C59R and S108N + *Pfdhps* A437G) confers partial resistance [9, 42, 43]. The quintuple mutant genotype (*Pfdhfr* N51I, C59R and S108N + *Pfdhps* A437G and K540E) defined as fully-resistant parasite, is strongly linked with SP-resistance [44]. Sextuple mutant genotypes (*Pfdhfr* N51I, C59R and S108N + *Pfdhps* A437G, K540E and A581G) also referred as super-resistant parasites have been associated with clinical and parasitological failure to SP therapy [45–47]. These genotypes observed in sub-Saharan Africa, are strongly selected by the use of SP and are considered to be predictive of IPTp and IPTi failure [41, 48]. Due to the relatively high prevalence of *Pfdhps* K540E and A581G mutations, the quintuple (*Pfdhfr* N51I, C59R and S108N + *Pfdhps* A437G and K540E) and sextuple (*Pfdhfr* N51I, C59R and S108N + *Pfdhps* A437G, K540E and A581G) mutated genotypes are highly prevalent in East Africa and infections with the sextuple genotype were associated with reduced birthweight [47]. In contrast, they are present at low frequencies in West and Central Africa where the *Pfdhps* K540E mutation remains uncommon. A new *Pfdhps* mutation, at position 431 (I431V), has recently been reported in Nigeria and Cameroon [9, 12, 49]. A recent study by Oguike *et al* showed an increase in the prevalence of this mutation from 0 to 6.5% between 2003 and 2008, and up to 46% in 2010 in Enugu, Nigeria [50]. suggesting that the I431V mutation is widespread throughout this part of Africa. The octal mutant genotype which combines the *Pfdhfr* N51I, C59R, S108N and *Pfdhps* I431V, S436A, A437G, A581G, A613S (IRN/VAGKGS) could be strongly associated with SP failure in Central Africa for which the efficacy of IPTp-SP may be limited [9]. However, its role in parasite resistance to SP has yet to be described [51].

Earlier studies carried out in Cameroon have reported the presence of almost all mutations described above [13, 52, 53]. However, unlike in other African countries, relatively few studies have been carried out on the evolution of drug resistance following the introduction of ACTs and chemoprevention interventions [4, 9, 10, 15, 54]. Moreover, almost all the data available are limited to forest zones (Central, South-East and North-East Regions) in Cameroon [53]. Thus, the profile of malaria resistance in savannah areas, such as in Tibati, is still unclear. To ensure efficient national malaria treatment policy and preserve the chemoprevention, it is crucial to regularly monitor the prevalence of SNPs linked to antimalarial drugs resistance across the country.

This study was designed to assess the prevalence of known drug resistance genotypes in the *Pfcrt*, *Pfmdr1*, *Pfdhfr* and *Pfdhps* genes of *P*. *falciparum* collected from two geographically and epidemiologically distinct areas of Cameroon in order to identify differences in trends of antimalarial drug resistance in Cameroon.

## Materials and methods

### Study sites and sample collection

**Fig 1:**
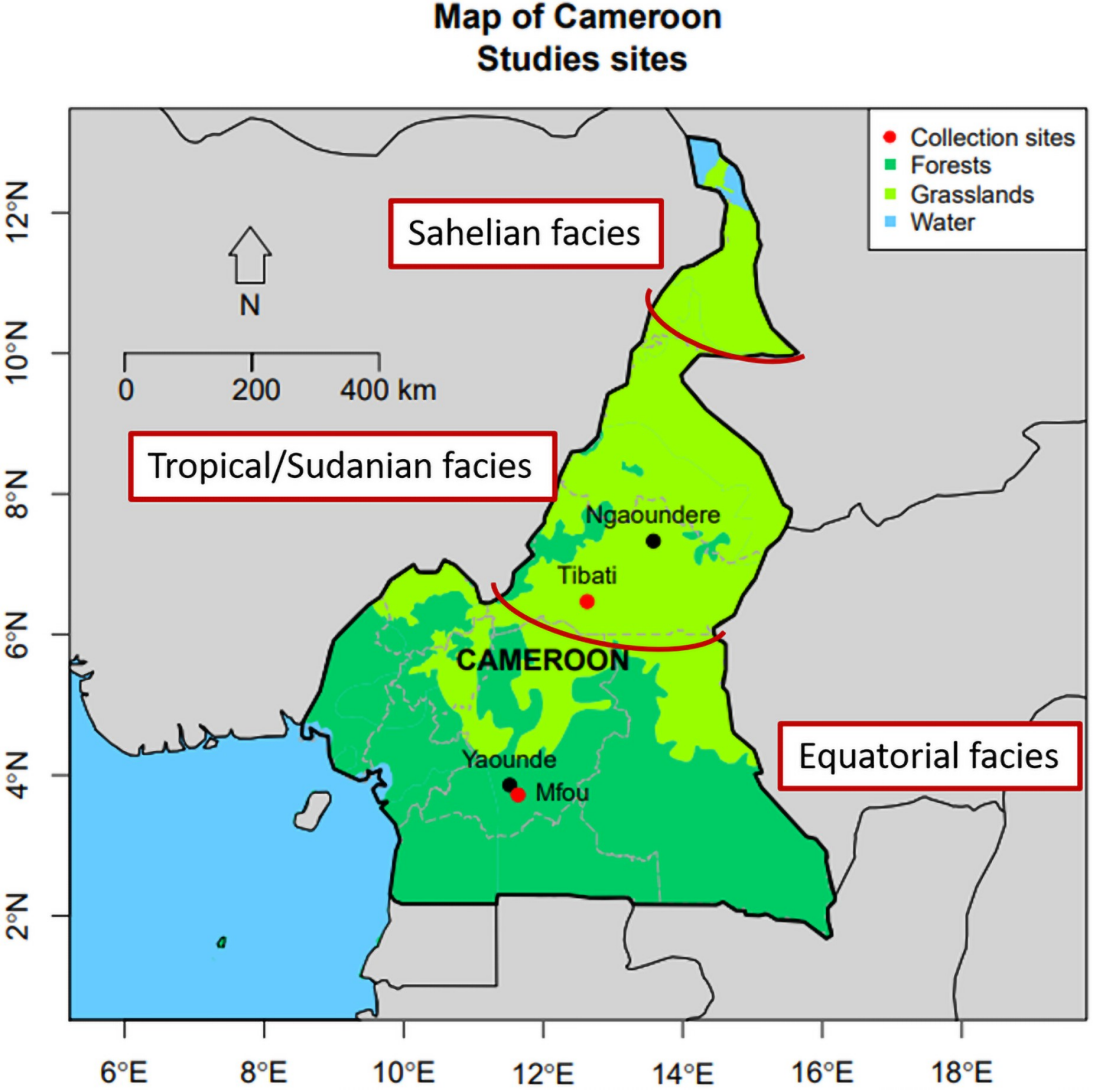
Map depicting the study sites in two different epidemiological settings of Cameroon. The map contains information from OpenStreetMap, which is made available under the Open Database License.

Cross-sectional studies were conducted between June—July 2018 in Mfou (3°72N; 11°64E) and July—August 2019 in Tibati (6°46’N, 12°63’E), two localities in Cameroon with different epidemiological settings (Fig 1).

Mfou is a semi-urban area located in the Central region of Cameroon and characterized by Guinea -type equatorial climate. Malaria transmission is perennial with higher prevalence during the rainy season from March to June and August to November. Tibati is located in the Adamawa region and characterized by humid savannah with one rainy season lasting over 6 months from March to October and seasonal transmission of malaria parasites. In both areas, *P*. *falciparum* is responsible for more than 90% of malaria infections, and *An*. *gambiae s*.*l* and *An*. *funestus* are the main malaria vectors [11].

In each study site, a simple random survey was carried out and participants were enrolled consecutively to limit selection and information bias. Prior to the survey, meetings with local authorities were organized to define suitable dates for collections in the different villages. Local populations were informed of the objectives and methods of the study during community sensitization surveys and were allowed to ask questions. Only individuals aged at least one year, with no fever (axillary temperature ≤ 37.5°C) the previous 48h, no ongoing antimalarial treatment and who have given their consent by signing an informed consent form were enrolled in the study. For minors, only those for whom parental consent was obtained were enrolled. Participation in the study was voluntary. Study participants were recruited among asymptomatic individuals in the communities. Asymptomatic carriers were defined as individuals with malarial parasitemia of any density, absence of fever or other acute malaria symptoms, no history of recent antimalarial treatment [55]. A semi-structured questionnaire was used to collect demographic data and information on the use of antimalarial drugs. Finger-prick blood samples were collected from each participant for rapid diagnostic test (RDT, SD Bioline) and thick blood smear microscopy diagnosis. Additionally, blood samples were spotted on a Whatman^®^ grade17 filter paper (Whatman^®^ Grade 17 Chr Cellulose Chromatography Paper, GE Healthcare, Chicago, USA), air-dried and stored in silica gel for further molecular analyses. The study participants who were found parasite-positive received free treatment with Artesunate Amodiaquine (ASAQ) according to the national guidelines by the National Malaria Control Program (NMCP).

### DNA extraction and molecular identification of *Plasmodium* species

Genomic DNAs from dried blood spots were extracted using the chelex-100 method as previously described [56] and stored at -20°C until molecular analyses. Detection and identification of *Plasmodium* spp were performed by real-time PCR according to Mangold *et al* [57]. The primer sequences target a polymorphic fragment of the small subunit 18S ribosomal RNA (18S rRNA) gene, allowing the differentiation of four plasmodial species, *P*. *falciparum*, *P*. *vivax*, *P*. *ovale* and *P*. *malariae*, upon analyses of the melting curves. Amplifications were run on a Light Cycler^®^ 96 instrument (Roche Molecular Systems, Indianapolis, USA) using SYBR Green dye for DNA detection and quantification.

### Single nucleotide polymorphisms (SNPs) associated with antimalarial resistance in *Pfcrt*, *Pfmdr1*, *Pfdhfr* and *Pfdhps* genes

Genotyping was performed for samples identified as *P*. *falciparum*-positive by real-time PCR. Nested PCR was then followed by restriction fragment length polymorphism (RFLP) analysis as described previously (University of Maryland School of Medicine. Available: https://www.medschool.umaryland.edu/malaria/Protocols/.) This allowed the detection of point mutations in *Pfcrt*, *Pfmdr1*, *Pfdhfr* and *Pfdhps* genes. The corresponding SNPs are detailed in Table 1.

**Table 1:** Characteristics of the primers used for PCRs and enzymes for ASRA.

Genes	Mutations	Primer names	Nucleotide sequence (5′–3′)	Annealing (°C)	Fragment size (bp)	Restriction enzyme	Fragment size (bp)
***Pfcrt***	**K76T**	76 F	ATG GCT CAC GTT TAG GTG GAG	**45**	192	**ApoI**	**136 + 56**
		76R	CGG ATG TTA CAA AAC TAT AGT TACC				
***Pfmdr1***	**N86Y**	86 F1	TTG AAC AAA AAG AGT ACC GCT G	**52**	450		
		86 R1	TCGTACCAATTCCTGAACTCAC				
		86 F2	TTT ACC GTT TAA ATG TTT ACC TGC	**52**	291	**ApoI**	**126 + 165**
		86 R2	CCA TCT TGA TAA AAA ACA CTT CTT				
	**D1246Y**	1246 F1	ATG ACA AAT TTT CAA GAT TA	**45**	295		
		1246 F2	ACT AAC ACG TTT AAC ATC TT				
		1246 F2	AAT GTA AAT GAA TTT TCA AAC C	**45**	203	**Bgl II**	**113 +90**
		1246 R2	CAT CTT CTC TTC CAA ATT TGA TA				
***Pfdhfr***	**S108N**	108 F1	ATG ATG GAA CAA GTC TGC GAC GTT TTC GA	**56**	580		
		108 R1	CAC ATT CAT ATG TAC TAT TT				
		108 F2	TAA TAA CTA CAC ATT TAG AGG	**56**	428	**Alu I**	**228 +200**
		108 R2	CTA AAA ATT CTT GAT AAA CAA CGG AAC CTC C				
	**I164L**	164 F1	CAGTTACAACATATGTGA	**45**	414		
		164 R1	CACATTCATATGTACTATTT				
		164F2	CTAATTCTAAAAAATTACAAAATGT	**45**	254	**Psi I**	**42 +212**
		164 R2	TTTCTTTTCTAAAAATTCTTGATAAACAACGGAACCTC**T**TA				
	**N51I /C59R**	51/59 F1	TAA TAA CTA CAC ATT TAG AGG	**45**	147		
		51/59 R1	ACA TCT CTT ATA TTT CAA TTT				
		51/59 F2	CTA GGA AAT AAA GGA GTA TTA CCA TGG AAA TG**G** A	**45**	113	**EcoRI**	**35+78**
		51/59 R2	ATT TTT CAT ATT TTG ATT CAT TCA CAT ATG TTG TAA CTG **T**AC			**BsrGI**	**43 + 70**
		437 F1	TAT TAA ATG TTA ATT ATG ATT CT	**52**	249		
***Pfdhps***	**A437G**	437 R1	TCA CAT TTA ACA ATT TTA TT				
		437 F2	TGT TCA AAG AAT GTT TGA AAT G	**52**	148	**AVAII**	**69 +79**
		437 R2	CCA TTC TTT TTG AAA TAA TTG				
	**K540E/A581G**	540/581 F1	AAA CAA ATT CTA TAG TG	**45**	256		
		540/581 R1	TGG ATA CTC ATC ATA TA				
		540/581 F2	GTT CTA ATG CAT AAA AGA GG	**45**	201	**FokI**	**56+ 145**
		540/581R2	TAA GAG TTT AAT AGA TTG ATC AGC TTT CTT C			**MwoI**	**36 +154**

F: Forward primer sequence; R: Reverse primer sequence.

Briefly, PCRs were carried out in a final volume of 25 μL containing 1X Hot-Start PCR Master Mix (Thermo Scientific, Waltham, MA, USA) and 10 nM of each primer (Eurogentec, Belgium). Purified DNA from the wild type (3D7) and multi-drug resistant laboratory strains (Dd2) of *P*. *falciparum* (MR4, ATCC^®^, Manassas VA, USA) were used as controls. Each assay included the two control DNA samples of *P*. *falciparum*: the CQ-susceptible strain 3D7 with the wild-type alleles *Pfcrt* K76, *Pfmdr1* N86 and D1246 and the CQ-resistant Dd2, carrying *Pfcrt* 76T, *Pfmdr1* 86Y and 1246Y mutant alleles. For *Pfdhfr* and *Pfdhps* mutations, 3D7 strain known to be sulfadoxine-pyrimethamine sensitive was included as control, the strain is carrying the wild alleles *Pfdhfr* S108, I164, N51, C59 and *Pfdhps* A437, K540 and A581. Primer pairs and cycling conditions are presented in Table 1. Digestion reactions were set at a final volume of 15 μL containing 8 μL of the nested PCR product, enzyme buffer and 1 unit of the appropriate restriction enzyme (Thermo Fisher Scientific). Mixtures were digested overnight at 37°C for all the restriction enzymes, as recommended by the manufacturer. A 7 μL volume of the digested product was loaded on a 2% agarose gel containing ethidium bromide and the gel was observed on a transilluminator with UV light. Restriction enzymes and fragment sizes for each point mutation are presented in Table 1.

### Data management and statistical analysis

Data were recorded in Microsoft Excel (Office 2010) and analyzed using the R Core Team (2020) and GraphPad Prism5 (San Diego, CA, USA) softwares. The site maps were drawn using R, maptools and maps packages with data source from OpenStreetMap. Information from OpenStreetMap is made available under the Open Database License. Graphics were built using ggplot2 package. Alleles corresponding to the *Pfdhfr/Pfdhps* SNPs were combined for each individual and genotypes recorded. Comparison of prevalence of infection and frequency of antimalarial resistance mutations between the two study areas was computed using the Chi-square and Fisher’s exact tests. The level of statistical significance was set at a value of P ≤ 0.05.

The sample size was calculated by the Lorentz formula: n = T2 x P (1-P) / M2 [58] where n = required sample size, T = 95% confidence level (standard value is 1.96), P = estimated prevalence of malaria in the study site (47% in Central region (Mfou) and 32% in Adamawa (Tibati) [59]), M = margin of error at 5% (standard value is 0.05), 1-P = adverse event.


$$\text{Mfou}:\text{n}={\text{1.96}}^{\text{2}}\text{x}\;\text{0.47}\left(1–0\;\text{.47}\right)/{\text{0.05}}^{\text{2}}\;\text{n}=\text{382.}\;\text{77}$$

$$\text{Tibati}:\text{n}=1{\text{.96}}^{\text{2}}\text{x}\;0\text{.32}\left(1–0\text{.32}\right)\text{/}\;\text{0}{\text{.05}}^{\text{2}}\;\text{n}=\text{334.}37$$

The minimum sample sizes were 383 and 334 individuals in Mfou and Tibati, respectively.

### Ethics statement

This study was reviewed and approved by the Cameroon National Ethics Committee for Research on Human Health (CNERSH, No. 2018/05/1011/CE/CNERSH/SP and CNERSH, No. 2019/05/1161/CE/CNERSH/SP).

## Results

### Characteristics of study population

Overall, 2,926 asymptomatic individuals were enrolled, 1,635 (55.88%) in Mfou and 1,291 (44.12%) in Tibati (Table 2).

**Table 2:** Basic characteristics of population survey.

	Both sites n = 2926	Mfou n = 1635	Tibati n = 1291	P-value
Sex/ratio, n (%)				
**Female**	1514 (51.74)	830 (50.76)	684 (52.98)	0.2333
**Male**	1412 (48.26)	805 (49.23)	607 (47.02)	
Median Age (years)	10	10	10	
Age Category n (%)				
**≤5 years**	663 (26.66)	337 (20.61)	326 (25.25)	0.1281
**6–10 years**	970 (33.15)	571 (34.92)	399 (30.91)	
**11–15 years**	680 (23.24)	439 (26.85)	241 (18.67)	
**16–20 years**	135 (4.61)	56 (3.42)	79 (6.12)	
**≥ 21 years**	478 (16.34)	232 (14.19)	246 (19.05)	

The age range of the participants from both sites was 1 to 95 years, with a median age of 10 years. No significant difference was observed for sex ratios in the two populations (P = 0.2333), nor for distribution over age groups (P = 0.1281).

**Fig 2:**
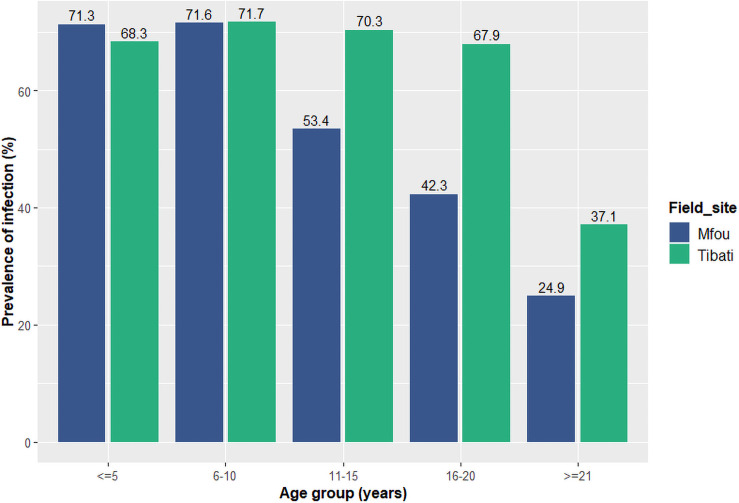
Distribution of *Plasmodium* infections among age groups in Mfou and Tibati. In Mfou, the infection prevalence steadily decreased by age group and reached 24.9% in the age group ≥ 21 years. In Tibati, the prevalence remained close to 70% in all age groups and only dropped in the age group ≥ 21 years to 37.1%.

Out of the 2926 samples collected, 2754 (1480 from Mfou, 1274 from Tibati) were successfully used for molecular analysis. The *Plasmodium*-specific real time PCR revealed 876/1480 (59.18%) and 808/1274 (63.42%) of positive isolates in Mfou and Tibati respectively. *P*. *falciparum* was the most prevalent *Plasmodium* species in both sites, accounting for >98% of infections, 839/876 (95.8%) and 791/808 (98%) in Mfou and Tibati respectively. *P*. *malariae* infections were identified only in Mfou, in 37/876 (4.2%) of the samples and 20/37 (54%) were mixed *Pf /Pm* infections. *P*. *ovale* was only found in Tibati in 17/791 (2%) of blood samples and 5/17 (29.4%) were mixed *Pf/Po* infections. All the 1,655 *P*. *falciparum* positive blood samples (859 from Mfou, 796 from Tibati) were genotyped for all molecular markers. The distribution of *Plasmodium* infections among the age groups is presented in Fig 2. Participants ≤10 years were the most infected, the malaria prevalence closed to 70% for this age category in both sites.

### Prevalence of genotypes in *Pfcrt* and *Pfmdr1* genes

Out of the 859 *P*. *falciparum* positive isolates from Mfou, 809 (94.18%), 827 (96.27%) and 826 (96.16%) were successfully genotyped for K76T, N86Y and D1246Y SNPs, respectively. Among the 796 *P*. *falciparum* positive isolates from Tibati, 762 (95.73%) for K76T, 739 (92.83%) for N86Y and 736 (92.46%) for D1246Y were genotyped. The prevalence of the *Pfcrt* and *Pfmdr1* genotypes are presented in Table 3.

**Table 3:** Frequency of *Pfcrt* and *Pfmdr1* genotypes in *Plasmodium falciparum* isolates from Mfou and Tibati, Cameroon.

Genes/Alleles	Number of samples (%)			
	Both sites	Mfou	Tibati	P value
***Pfcrt* codon 76**				
***N samples processed***	**1571**	**809**	**762**	
Wild-type K76	1244 (79.18)	716 (88.50)	528 (69.29)	< 0.0001^a^
Mixed K76 and 76T	198 (12.60)	70 (8.65)	128 (16.80)	
Mutant 76T	129 (8.21)	23 (2.84)	106 (13.91)	
**Total mutant 76T**	**327(20.81)**	**93(11.50)**	**234(30.71)**	< 0.0001^b^
***Pfmdr1* codon 86**				
***N samples processed***	**1566**	**827**	**739**	
Wild-type N86	769 (49.11)	369 (44.62)	400 (54.13)	< 0.0001^a^
Mixed N86 and 86Y	605 (38.63)	381 (46.07)	224 (30.31)	
Mutant 86Y	192 (12.26)	77 (9.31)	115 (15.84)	
**Total mutant 86Y**	**797(50.89)**	**458(55.38)**	**339(45.87)**	0.0002^b^
***Pfmdr1* codon 1246**				
***N samples processed***	**1562**	**826**	**736**	
Wild-type D1246	1501 (96.09)	809 (97.94)	692 (94.02)	< 0.0001^a^
Mixed D1246 and 1246Y	49 (3.13)	8 (0.97)	41 (5.57)	
Mutant 1246Y	12 (0.77)	9 (1.09)	3 (0.46)	
**Total mutant 1246Y**	**61(3.91)**	**17(1.45)**	**44(5.97)**	< 0.0001^b^

The distribution of alleles at each SNP was compared between Mfou and Tibati using a Chi square test and the P-value is indicated.

^a^, comparison of wild-type, mixed and mutant genotypes.

^b^, comparison of total mutant versus wild-type genotypes.

The frequency of the *Pfcrt* K76 wild type allele was 88.50% (716/809) and 69.29% (528/762) respectively. The *Pfcrt* 76T mutant allele was observed in 23/809 (2.84%) and 106/762 (13.91%) from Mfou and Tibati, respectively. Mixed *Pfcrt* alleles were identified in 70/809 (8.65%) and 128/762 (16.80%) of the blood isolates, in Mfou and Tibati, respectively. There was a significant difference in the distribution of the alleles between the two studied sites, when we compared wild-type, mixed and mutant genotypes (P < 0.001) as well as when mixed genotypes were classified in the mutant group (P < 0.001). The frequencies of the wild type allele *Pfmdr1* N86 were 44.62% (369/827) and 54.13% (400/739) in Mfou and Tibati respectively. The mutant allele *Pfmdr1* 86Y was found in 77/739 (9.31%) and 115/739 (15.84% of the samples in Mfou and Tibati, respectively.). Mixed (86 N/Y) *Pfmdr1* alleles identified in 381/827 (30.31%) and 224/739 (46.07%) of the isolates in Mfou and Tibati, respectively.

Statistically significant differences were observed when comparing wild-type, mixed and mutant genotypes (P < 0.001) and wild-type *versus* total mutant genotypes (P = 0.002). The *Pfmdr1* N86 + *Pfcrt* 76K (NK) genotype, associated to a higher susceptibility to lumefantrine (L), was found in 317/827 (40.64%) and 251/739 (37.92%) isolates in Mfou and Tibati, respectively and the difference is not significant (χ2 = 3.21, P = 0.073). The *Pfmdr1* D1246 wild type allele was detected in 809/826 (97.94%) and 692/736 (94.02%) of the isolates from Mfou and Tibati, respectively. The mutant-type 1246Y was present in 9/826 (1.09%) and 3/736 (0.46%) of isolates from Mfou and Tibati, respectively. Mixed alleles (1246 D/Y) were scored in 8/826 (0.97%) samples in Mfou and 41/736 (5.6%) in Tibati.

### Prevalence of SNPs in *Pfdhfr* and *Pfdhps* genes

The results of the allele-specific restriction analyses for each codon are presented in Table 4. Nearly all samples contained the *Pfdhfr* mutations at codons 51, 59 and 108. The overall prevalence of the mutant allele 51I was 1565/1619 (96.66%), with the occurrence being significantly more frequent in isolates from Mfou 818/825 (99.15%) than those from Tibati 747/794 (94.08%); P < 0.0001. The frequency of the mutant allele 59R was higher in Mfou, 827/833 (99.28%) than Tibati 773/794 (97.36%), (P = 0.0024). The 108N allele appeared in all samples from Mfou (821) and in 779/783 (99.49%) isolates from Tibati. The difference was significant (P = 0.0403), and the overall prevalence was 1600/1604 (99.7%). For 164 codon, all parasite isolates harbored the wild type allele I164. we combined the different SNPs for the *Pfdhfr* gene, and the triple mutant 51I, 59R, 108N (IRN) was the most frequent combination, found in 763/772 (98.83%) and 674/724 (93.09%) of samples in Mfou and Tibati, respectively (χ2 = 32.49, P < 0.0001).

**Table 4:** Frequency of *Pfdhfr* and *Pfdhps* genotypes in *Plasmodium falciparum* isolates from Mfou and Tibati, Cameroon.

Genes	Codons/alleles	Number of samples (%)			
		Both sites	Mfou	Tibati	P value
*Pfdhfr*	**Codon 51** *N samples processed*	**1619**	**825**	**794**	
	Wild-type N51	54 (3.33)	7 (0.85)	47(5.92)	<0.0001
	Mutant-type 51I	1565 (96.66)	818 (99.15)	747(94.08)	
	**Codon 59** *N samples processed*	**1627**	**833**	**794**	
	Wild-type C59	27 (1.65)	6(0.72)	21(2.64)	0.0024
	Mutant-type 59R	1600 (98.34)	827 (99.28)	773(97.36)	
	**Codon 108** *N samples processed*	**1604**	**821**	**783**	
	Wild-type S108	4 (0.23)	0(0)	4(0.51)	0.0403
	Mutant-type 108N	1600 (99.75)	821(100)	779(99.49)	
	**Codon 164** *N samples processed*	**1604**	**824**	**785**	
	Wild-type I164	1609 (100)	824(100)	785(100)	NA
	Mutant-type 164L	0 (0)	0 (0)	0(0)	
*Pfdhps*	**Codon 437** *N samples processed*	**1579**	**809**	**770**	
	Wild-type A437	147(9.31)	42(5.19)	105(13.64)	<0.0001
	Mutant-type 437G	1432(90.69)	767 (94.80)	665(86.36)	
	**Codon 540** *N samples processed*	**1565**	**811**	**754**	
	Wild-type K540	1560(99.68)	810(99.88)	750(99.47)	0.2023
	Mutant-type 540E	5 (0.32)	1(0.12)	4(0.53)	
	**Codon 581** *N samples processed*	**1566**	**811**	**755**	
	Wild-type A581	1515 (96.74)	796 (98.15)	719(95.23)	0.001
	Mutant-type 581G	51 (3.26)	15 (1.85)	36 (4.77)	

The distribution of alleles at each SNP was compared between Mfou and Tibati using a Chi square test and the P-value is indicated. NA, not applicable.

**Fig 3:**
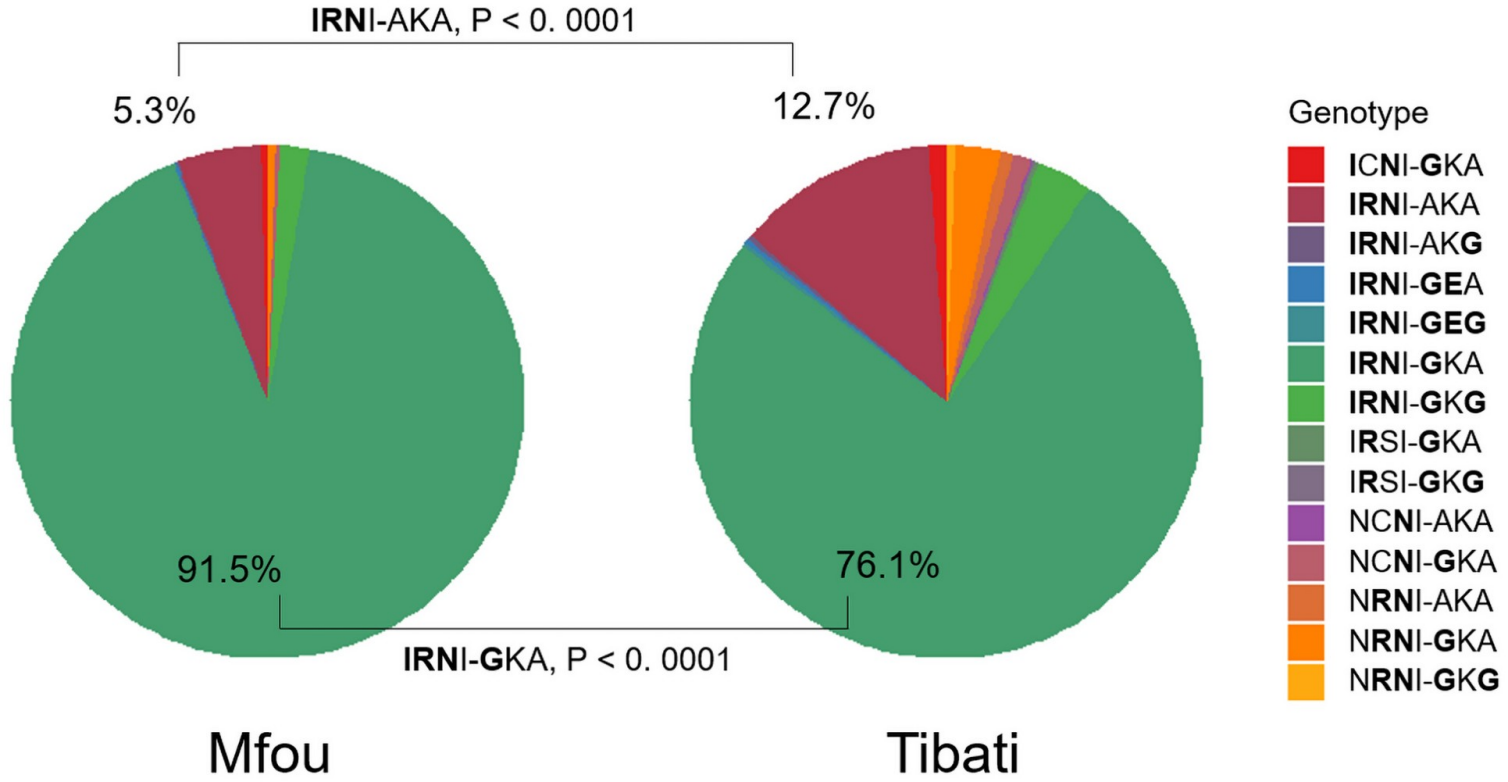
Prevalence of combined *Pfdhfr-Pfdhps* genotypes in Mfou and Tibati. The quadruple mutant **IRN**I-**G**KA was the most prevalent in both study sites, 706/772 (91.45%) in Mfou and 552/724 (76.24%) in Tibati, and the difference is significant (χ2 = 64.59, P < 0.0001; Fig 3). The triple mutant **IRN**I-AKA was more frequent in Tibati (12.7% versus 5.3% in Mfou, χ2 = 24.98, P < 0.0001; Fig 3). The wild-type genotype NCSI-AKA was never identified. The super-resistant genotype **IRN**I-**GEG**, a sextuple mutant associated to high level of SP resistance appeared in two samples from Tibati.

In the *Pfdhps* gene, the 437G mutant allele appeared in more than 90% of the total isolates analyzed. It was found in 767/809 (94.80%) of the isolates from Mfou and 667/770 (86.36%) from Tibati (P < 0.0001). *Pfdhps* mutations K540E and A581G were present in both sites although at low frequencies. We found the mutant allele 540E in five samples, 1/811 (0.12%) in Mfou and 4/754 (0.53%) in Tibati. The prevalence of the 581G mutant allele was 15/811 (1.85%) and 36/755 (4.77%) respectively, with statistically significant difference between both areas (P = 0.0011). The combination of the different mutations found in *Pdhfr* and *Pdhps* genes are presented in Fig 3. A total of 14 genotypes were observed and the distribution of the different genotypes differed between the two study sites (P <0.0001). A higher diversity was observed in Tibati with 14 genotypes whereas, only 8 genotypes were recovered among samples from Mfou.

## Discussion

Antimalarial drug resistance remains a challenge for malaria control and elimination in most endemic countries. Regular surveillance of *P*. *falciparum*resistance is essential to monitor the efficacy of treatment guidelines. This study assessed the prevalence of antimalarial drug resistance mutations in *P*. *falciparum* genes, *Pfcrt*, *Pfmdr1*, *Pfdhfr* and *Pfdhps*, in two different regions of Cameroon (Central and Adamawa Regions) more than 15 years after the malaria drug policy have changed from the use of CQ through AQ and SP to ACTs in 2004 [8, 53].

The present study found a high prevalence of the *Pfcrt* K76 wild type genotype in our sample sets (79.18%). This finding is in line with earlier studies conducted in the Northwest Region of Cameroon [60], in the Eastern part of Cameroon [54] and in Southwest Region [12], that also reported the circulation of chloroquine-sensitive strains several years after CQ withdrawal as first-line treatment for malaria. The re-emergence of *P*. *falciparum* carrying the wild type allele was first observed in Malawi [61] and then in other African countries such as Tanzania [62], Kenya [63], Burkina Faso [35] and Ivory coast [64]. Despite the fact that the Cameroon government officially banned the use of chloroquine in 2002 [7], the frequency of the K76 wild type within the country was low until recently and differed between epidemiological settings [54, 65]. Indeed, the prevalence of *Pfcrt* K76 wild type reported in this study is higher than the 53% reported by Ndam *et al*. [54] in samples collected in 2012 in the Eastern part of Cameroon. This suggests that the return of chloroquine sensitivity may not be similar across the country, possibly because of different drug usage. Also, samples in Ndam’s study were collected 9 years following the change in treatment policy whereas our study was carried out 15 years later. Our results then confirm that the decline in CQ resistant genotypes takes longer in West and Central Africa than in East Africa [66]. Here, we found a significantly higher proportion of the K76 wild type allele in Mfou than in Tibati (88.50% vs 69.29%, P < 0.0001) and the difference could be due to the different use of CQ in the community. Indeed, despite the official withdrawal Of CQ in 2002, the drug is still available through informal outlets, and particularly used for self-medication in some rural areas such as Tibati [15]. In 2012, a household survey conducted in Nigeria found that more than 50% of children suffering from malaria were given CQ even though the national malaria drug policy had changed to ACTs seven years ago [67, 68]. Hence, this CQ drug pressure has potentially interfered on recovery of CQ sensitive parasites in Nigeria. It is possible that CQ is also sold on local markets in Tibati because of its proximity with Nigeria or alternatively, the cross-border movements of people allow the spread [68, 69]. High level of the *Pfcrt* 76T mutant (74,6%) was observed in 2019 along the Cameroon-Nigeria border area [68], which further supports the above explanations. In addition, the use of SPAQ combination for SMC in the northern regions of Cameroon may lead to a selective pressure on the *Pfcrt* 76T mutant allele, as AQ resistance has been associated with selection of *Pfcrt* 76T and *Pfmdr* 86Y [33]. By contrast, ACTs may be more accessible for people living in Mfou, due to its vinicity with the capital city, Yaoundé, and a larger use of AL for the treatment of uncomplicated malaria in Mfou may have contributed to the selection for the K76 allele [27].

The mutant allele 86Y in the *Pfmdr1* gene is associated with CQ resistance [70]. In the early 2000, the prevalence of *Pfmdr1* 86Y mutant allele was over 80% in several regions of Cameroon [13–15]. Our work shows a decrease in the prevalence of *Pfmdr1* 86Y mutant alleles, reaching 55.38% and 45.87% respectively in Mfou and Tibati. The results further show a low prevalence of *Pfmdr1* 1246Y mutant alleles (1.45 and 5.97%, respectively), a mutation related to CQ and quinine resistance. Our results are consistent with previous studies in Cameroon [10, 12], Gabon [71, 72], Nigeria [73], Mozambique [25] and Uganda [74, 75], showing significant reduction of *Pfmdr1* 86Y and 1246Y mutant alleles after replacement of CQ by ACTs. However, an overall similar proportion of both alleles N86 and 86Y (49.11% vs 50.89%) was observed in this study. It is plausible that the concurrent distribution of mutant and wild-type allele at codon 86 is a result of the simultaneous use of AS-AQ (first line) and AL (second line) for treatments of uncomplicated malaria in Cameroon [7]. In addition, previous studies have reported that the presence of the *Pfmdr1* N86 and 86Y alleles is currently being driven by an ACT-linked *P*. *falciparum* opposite selection [26, 33] and that AL and AS-AQ exerted opposite trends in selecting *Pfmdr*1 N86Y and D1246Y mutations [26]. Following adoption of ACTs in many African malaria countries, some studies have shown that *Pfmdr1* 86Y and 1246Y mutant alleles are strongly selected by ASAQ [27, 76]. Other studies indicate that the N86 and D1246 wild-type alleles are linked to a decrease of susceptibility to AL [27, 77]. In regard of these findings, it may be advantageous to use the two ACTs at the same time in the population in order to reduce the selection pressure of each treatment line and ensure the continuous effectiveness of ACTs in Cameroon.

SP was used for the treatment of uncomplicated malaria in Cameroon before its implementation for IPTp. Previous studies reported circulation of SP resistant parasites in the Central, North and South-East regions in the early 2000s [4, 14]. One of the goals of this study was to determine the prevalence of *Pfdhfr* and *Pfdhps* genotypes associated with SP resistance at present. Mutations at codons 51, 59 and 108 on the *Pfdhfr* gene were near saturation in our study. Even though high prevalences of *Pfdhfr* resistance alleles among *P*. *falciparum* in Cameroon was were reported as early as 2000 [53], it is surprising to find high proportions nowadays despite the adoption of ACTs for treatment of uncomplicated malaria. It is possible that the SP combination is still widely used outside the official sector for treatment of uncomplicated malaria by people who prefer to revert to SP instead of the three days daily doses of ACTs [10]. Additionally, the large-scale deployment of the IPTp and SMC interventions has certainly contributed to increase of drug pressure which has driven the spread of parasite resistance to SP [53]. The triple mutant IRN associated with high-level of pyrimethamine resistance was found in most of the samples analyzed in this study. This finding is in agreement with recent reports from Cameroon [9, 12] and in line with other studies carried out in other African countries such as Gabon (100%) [78] and Nigeria (93%) [79]. The *Pfdhfr* 164L mutant allele has been shown to be strongly associated with high pyrimethamine resistance in East Africa. In the current study, the *Pfdhfr* 164L mutant allele was not detected, and this result is consistent with previous reports in Cameroon [9, 80]. This mutation was recently detected in Equatorial Guinea although at low frequency, only two samples harboring it, which suggests that the I164L mutation is not widespread in Central Africa [81].

Considering sulphadoxine resistance, the *Pfdhps* 437G mutant allele was observed in more than 90% of analyzed samples, and was significantly more frequent in samples from Mfou than Tibati (94.80% vs 86.36%; P < 0.0001). This prevalence was similar to that found by *Berzosa et al*. in Equatorial Guinea [81], but significantly higher than those reported few years ago in pregnant women from Yaoundé (76%) [9]. The current work showed a remarkable increase in 437G prevalence in Cameroon. It is known that the A437G mutation is strongly associated with resistance to sulfadoxine and increased risk of SP treatment failure in Africa [79]. The rising trend of the *Pfdhps* mutation underscores the need for regular monitoring in different areas of Cameroon where SP is used for malaria interventions. Although a higher prevalence of *Pfdhps* 437G mutant allele was observed in the present study. The prevalence of the *Pfdhps* mutant allele 540E was very low, detected in only 5 samples (0.33%). Hence, the prevalence of the *Pfdhps* 540E mutation in Cameroon remains low and relatively stable [9, 14]. In contrast, the *Pfdhps* 540E is common in East Africa, and its prevalence is generally high [41, 46]. This mutation is known to be less prevalent in Central Africa, which was confirmed in this study. Similar to 540E, 581G *Pfdhps* mutation has been reported to occupy the crucial position in the modulation of sulfadoxine resistance. In Cameroon, the *Pfdhps* 581G mutant allele was previously found in 5.9% of samples collected from Yaounde in 2015 [9]. Overall, this work reported a prevalence of 3.26% of the 581G allele. A meta- analysis in Africa demonstrated that IPTp efficacy was reduced when the prevalence of 581G was above 10% [82]. This threshold was not observed in this work. Although we do not report information on the *Pfdhps* I431V mutation in this study, it is important to note that this mutation is emerging in West and Central Africa, and has already been reported in Cameroon by Chauvin *et al*. and in Nigeria by Oguike *et al* [9, 50]. Its association with other *Pfdhps* mutations such as A437G and A581G identified here could be associated with SP failure. Further work will have to determine I431V prevalence in the studied areas.

The quadruple mutant *Pfdhfr* 51I/59R/108N + *Pfdhps* 437G (IRNG) conferring partial resistance to SP was most predominant in this study, with a prevalence close to 90%, and higher than reports from Equatorial Guinea (54%) [81] and Nigeria (42,6%) [79]. This high prevalence of quadruple mutants suggests high risks of SP treatment failure in IPTp and SMC. Mutations *Pfdhps* K540E and A581G are key indicators for the quintuple and sextuple mutants associated to SP failure [40, 41]. In 2015, the quintuple mutant that includes the *Pfdhps* 540E mutation was found in two samples from Yaounde in women who had taken SP in IPTp [9].

We found the *Pfdhps* 540E mutation in five samples as quintuple mutants carrying the IRNGE genotype, and this confirms the circulation of parasites fully resistant to SP in Cameroon. Another important finding of this work was the presence of the IRNGEG sextuple mutant genotype in 2 samples from Tibati, a genotype determining super resistant parasites and associated with SP treatment failure. Even if the effectiveness of SP in IPTp and SMC seems to be preserved in Cameroon, the fact that we have identified fully and super resistant parasites raises concerns about the future efficacy of SP chemoprevention. This finding therefore warrants thorough monitoring of *Pfdhps* and *Pfdhfr* mutation prevalence in Cameroon.

However, this survey was limited to two regions of Cameroon. Additional, and more extensive studies in other geographical parts of the country will be able to provide the best mapping tool for monitoring the spread and evolution of antimalarial drug resistance. Moreover, the molecular profile of antimalarial drug resistance should also target additional SNPs lacking in this study such as the *Pfmdr1* Y184F linked to modulation of ACTs sensitivity, *Pfdhps* I431V associated to SP resistance, and the *Pfk*13 SNPs related to artemisinin resistance.

## Conclusion

This work showed a high prevalence of the *Pfcrt* K76, *Pfmdr1* N86 and D1246 wild-type alleles in *P*. *falciparum* isolates from Mfou and Tibati, Cameroon, indicating a return of the 4-aminoquinoline susceptible parasite strains, especially to CQ sensitive genotypes. This finding is in line with other reports from sub-Saharan Africa, even if return to CQ wild type parasites arose slowly in Central Africa. It has been postulated that re-emergence of CQ sensitive parasites raises the possibility of reintroducing CQ, in combination with another antimalarial drug, for malaria prevention and treatment. However, CQ drug pressure since the COVID-19pandemic in early 2020 is likely to change the current trend of CQ resistance decline. Furthermore, we report a high prevalence of *Pfdhfr* and *Pfdhps* mutant alleles conferring resistance to SP. The prevalence of *Pfdhfr* triple mutations was nearly saturated and there was an increased prevalence in *Pfdhps* 437G mutation when compared with previous reports in Cameroon and in other endemic countries. Unfortunately, partially, fully and super resistant genotypes conferring SP failure were detected in this study and this raises concerns about efficacy and future use of IPTp and SMC in Cameroon. Here, we provided the first report on the current situation of antimalarial drug resistance in the Adamawa region of Cameroon since the change of malaria drug policy. Globally, findings from this study indicate that increased vigilance and regular monitoring of molecular markers associated with malaria drug resistance will be crucial for malaria control in Cameroon.

## Abbreviations

**ACT**: Artemisinin-based combination therapy
**AL**: Artemether-Lumefantrine
AQ: Amodiaquine
**AQ-SP**: Amodiaquine-Sulphadoxine-Pyrimethamine
**AS**: Artesunate
**AS-AQ**: Artesunate-Amodiaquine
**CQ**: Chloroquine
**HL**: Halofantrine
**IPTp-SP**: Sulphadoxine–Pyrimethamine Intermittent preventive treatment for pregnant women
**Nested PCR**: Nested polymerase chain reaction
***Pfcrt***: *Plasmodium falciparum* chloroquine resistance transporter
***Pfdhfr***: *Plasmodium falciparum* dihydrofolate reductase
***Pfdhps***: *Plasmodium falciparum* dihydropteroate synthase
***Pfmdr1***: *Plasmodium falciparum*: multidrug resistance gene 1
**qPCR**: Quantitative polymerase chain reaction
**SMC-SP**: Sulphadoxine–Pyrimethamine Seasonnal Malaria Chemoprevention
**SNP**: Single-nucleotide polymorphism
**SP**: Sulphadoxine-Pyrimethamine
